# A device for the controlled cooling and freezing of excised plant specimens during magnetic resonance imaging

**DOI:** 10.1186/s13007-021-00743-4

**Published:** 2021-04-13

**Authors:** Camilo Villouta, Benjamin L. Cox, Beth Rauch, Beth Ann A. Workmaster, Kevin W. Eliceiri, Amaya Atucha

**Affiliations:** 1 Department of Horticulture, University of Wisconsin-Madison, 1575 Linden Dr., Madison, WI 53706 USA; 2 Medical Engineering Group, Morgridge Institute for Research, 330 N Orchard St, Madison, WI 53706 USA; 3 Laboratory for Optical and Computational Instrumentation (LOCI), University of Wisconsin-Madison, 1675 Observatory Dr., Madison, WI 53706 USA; 4 Department of Medical Physics, University of Wisconsin-Madison, 1111 Highland Ave., Madison, WI 53705 USA; 5 Department of Biomedical Engineering, University of Wisconsin-Madison, 1415 Engineering Dr., Madison, WI 53706 USA

**Keywords:** MRI, 3D printing, Dormant bud, Freezing resistance, Plant cold hardiness

## Abstract

**Background:**

Investigating plant mechanisms to tolerate freezing temperatures is critical to developing crops with superior cold hardiness. However, the lack of imaging methods that allow the visualization of freezing events in complex plant tissues remains a key limitation. Magnetic resonance imaging (MRI) has been successfully used to study many different plant models, including the study of in vivo changes during freezing. However, despite its benefits and past successes, the use of MRI in plant sciences remains low, likely due to limited access, high costs, and associated engineering challenges, such as keeping samples frozen for cold hardiness studies. To address this latter need, a novel device for keeping plant specimens at freezing temperatures during MRI is described.

**Results:**

The device consists of commercial and custom parts. All custom parts were 3D printed and made available as open source to increase accessibility to research groups who wish to reproduce or iterate on this work. Calibration tests documented that, upon temperature equilibration for a given experimental temperature, conditions between the circulating coolant bath and inside the device seated within the bore of the magnet varied by less than 0.1 °C. The device was tested on plant material by imaging buds from *Vaccinium macrocarpon* in a small animal MRI system, at four temperatures, 20 °C, − 7 °C, − 14 °C, and −  21 °C. Results were compared to those obtained by independent controlled freezing test (CFT) evaluations. Non-damaging freezing events in inner bud structures were detected from the imaging data collected using this device, phenomena that are undetectable using CFT.

**Conclusions:**

The use of this novel cooling and freezing device in conjunction with MRI facilitated the detection of freezing events in intact plant tissues through the observation of the presence and absence of water in liquid state. The device represents an important addition to plant imaging tools currently available to researchers. Furthermore, its open-source and customizable design ensures that it will be accessible to a wide range of researchers and applications.

## Introduction

Woody plants' adaptations to withstand freezing temperatures are key for their survival in environments with extreme winter temperatures, as well as, in the case of agricultural crops, for the attainment of consistent yields under the threat of climate change [1–4]. A better understanding of the freezing survival mechanisms utilized by plant tissues is critical to identifying genetic variation in freezing tolerance, and thus the development of plant material with enhanced cold hardiness.

Woody plant buds are particularly vulnerable to damage by extreme freezing temperatures [5]. Although several studies in multiple woody species have focused on freezing patterns and freezing tolerance of buds [6–8], the technical difficulties of visualizing freezing events of individual structures within woody buds remain a major limitation to this line of research [7].

Currently, magnetic resonance imaging (MRI) is the only tool with which the non-destructive study of the propagation and distribution of ice formation in complex plant organs at different experimental temperatures is possible [9]. The use of this technique is based on the drastic change in signal intensity detected by MRI when water changes from liquid state to solid state (ice). It is possible to analyze MRI images by measuring mean gray values (MGV), where bright areas represent liquid water, and dark areas represent areas of low proton density, as well as water that has just frozen [10]. Insight into the freezing mechanisms used by plant tissues can be determined by the acquisition of images at a series of different temperatures [7], allowing researchers to infer where and when ice is being formed. This approach results in a robust technique for the study of plant cold hardiness [11].

MRI has been successfully used to study many different plant models, including the study of in vivo changes during freezing for several species [6–8, 11, 12], physiological processes [13], and dormancy induction in woody plants, including their adaptation to environmental conditions [14, 15]. However, despite its benefits and past successes, the use of MRI in plant sciences remains low, likely due to limited access, high costs, and associated engineering challenges. Engineering challenges are of particular concern when attempting the study of plant buds with MRI at freezing temperatures, given the need to keep plant samples at consistent freezing temperatures within the MRI equipment for many hours. Addressing these challenges, the objective of this report is to describe the design, fabrication, and testing of a low-cost MRI-compatible sample holder and cooling device that enables the controlled freezing of plant specimens while samples are scanned in an MRI device.

## Results and discussion

### Device design

**Fig. 1:**
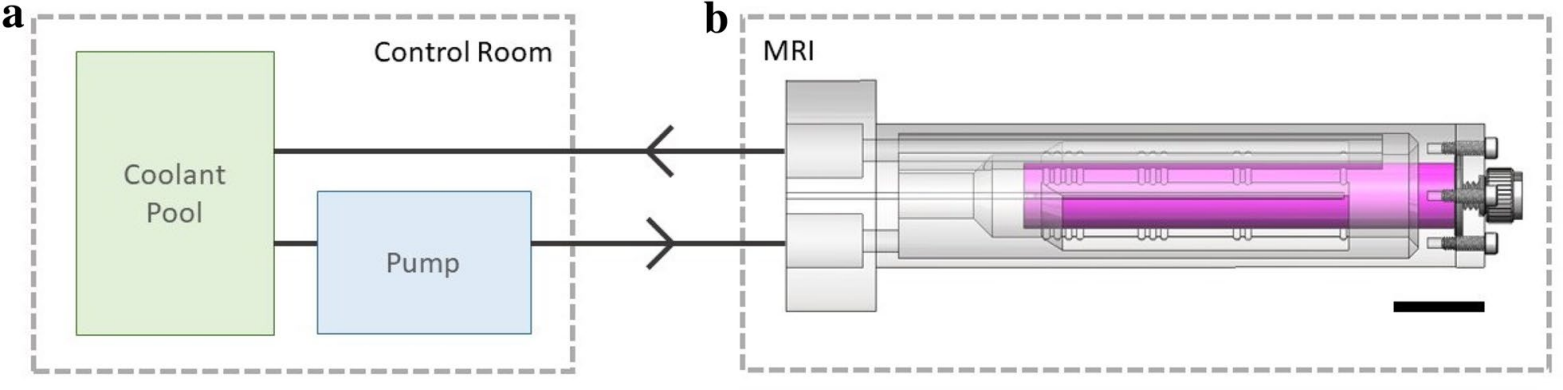
Cooling circuit and device design. Schematic showing the cooling circuit detailing locations of the coolant pool and pump in the control room (**a**) and the cooling device within the MRI (**b**). CAD diagram of assembled cooling device (**b**) highlighting the sample chamber (pink). The remaining interior volume is comprised of the surrounding coolant chamber through which chilled ethylene glycol is pumped. Scale bar is approximately 2 cm

**Fig. 2:**
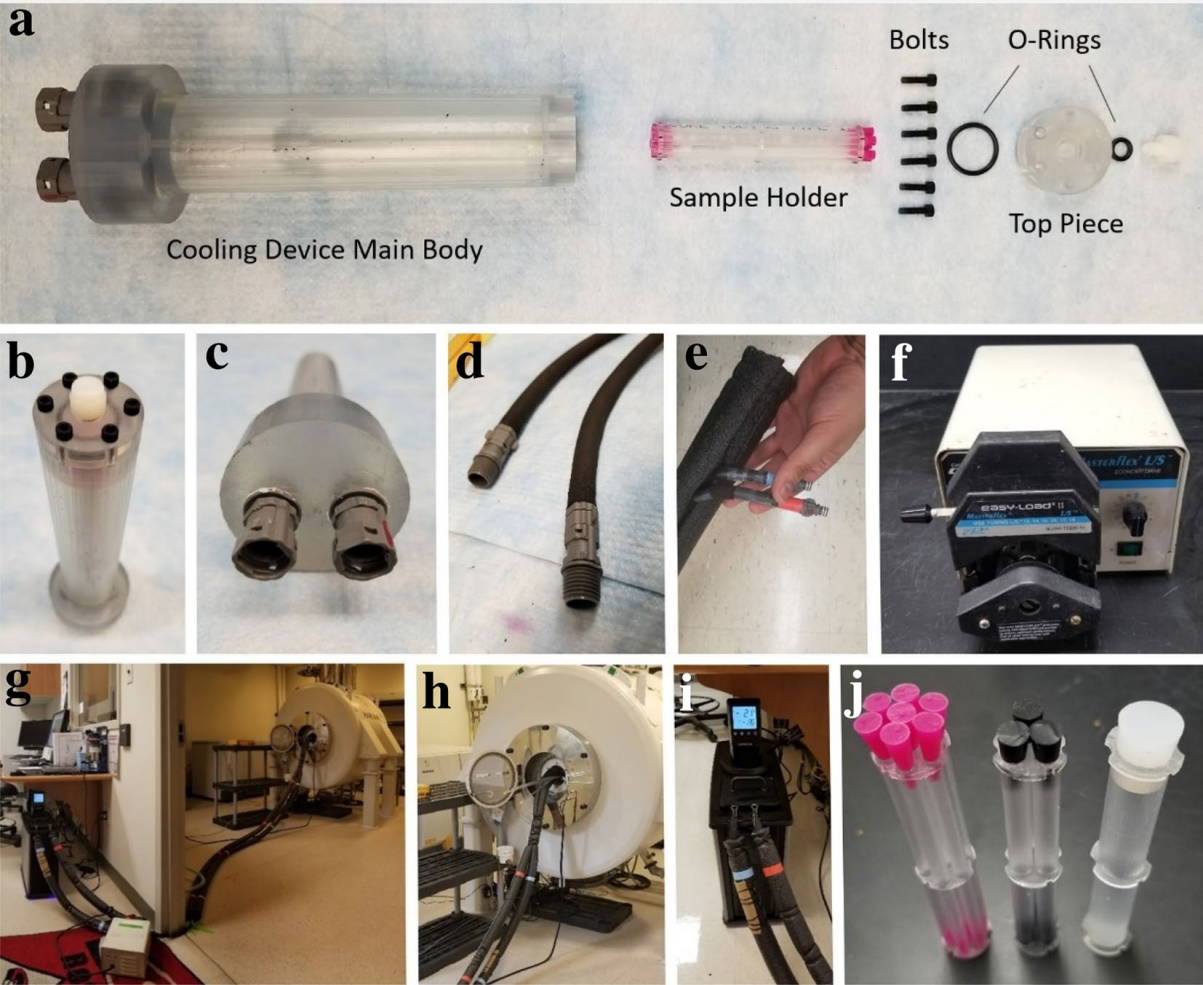
Components of the cooling device and experimental cooling circuit. The fabricated cooling device (**a**–**c**), including the individual device parts (**a**), view of cap end of the assembled device (**b**), and view of coolant inlet/outlet end of the assembled device (**c**). Neoprene-insulated Tygon® tubing, for conducting the chilled ethylene glycol (**d**), is further wrapped in an additional layer of polyethylene pipe insulation (**e**). A peristaltic pump (**f**) delivers the chilled ethylene glycol to the cooling device while situated in the MRI unit (**g**, **h**) from the circulating bath (coolant pool) (**i**) via the insulated tubing. Chilled coolant flows through the sample chamber, containing the sample holder comprised of several individual sample tubes. Sample holders were made to hold tubes of three different sizes (**j**), to accommodate a range of excised plant specimens

The overall assembly and individual components of the cooling device were designed using SolidWorks (Dassault Systemes, Velizy-Villacoublay, France), a computer-aided design (CAD) software package. The resulting device was a custom assembly to hold the samples and sit within the bore of the magnet. The device was part of a cooling circuit that includes a temperature-controlled coolant pool and pump located in the control room of the MR system, as well as tubing and fittings to connect the two sides of the circuit (Fig. 1). The assembled device (Fig. 1b) consisted of a coolant chamber surrounding a sample chamber that are independent of one another. The independence of the inner sample chamber is for the use of an inert perfluorocarbon solution typically used to enhance MR image clarity (Fig. 1b). Cooled coolant was pumped through the coolant chamber and around the sample chamber, effecting heat transfer from the plant specimen within the sample chamber (Fig. 1a). The individual components of the device included three custom 3D printed parts and several commercially available components (Table 2). Three interchangeable sample holders were designed and built (Fig. 2j) for flexibility with a range of plant specimens.

### Device fabrication

There were three custom components of the cooling device (Fig. 2), which were 3D printed using a Viper Si2 Stereolithography System (3D Systems, Rock Hill, SC, USA) using Accura60, a proprietary photopolymer resin (3D Systems). This resin has chemical similarities to UV-cure epoxy, requiring the use of UV light for solidification. This material, when solid, has similar properties to acrylic. The main body of the device was printed upright with the larger end facing down and without supports on the inside of the coolant chamber (to avoid supports being trapped inside). The larger holes connected to the coolant chamber (Fig. 2a, left side) were tapped to accommodate the fittings used to pump the coolant. The smaller holes at the other end of the coolant chamber (Fig. 2a, right side) were tapped for bolts to seal the device cap. A 2 mm diameter conduit was made from the larger end and until the base of the inner chamber to place a temperature probe. Lastly, the smallest custom component, the cap, was used to seal the sample chamber using an O-ring. A center hole in the cap can be sealed last, after assembly, and is used to top off the fluid in the sample chamber. All materials, tubing connectors, and bolts were selected to be MRI-compatible (non-ferromagnetic and low magnetic susceptibility), and to not interact with the circulating coolant (Fig. 2).

### Novel design features

**Fig. 3:**
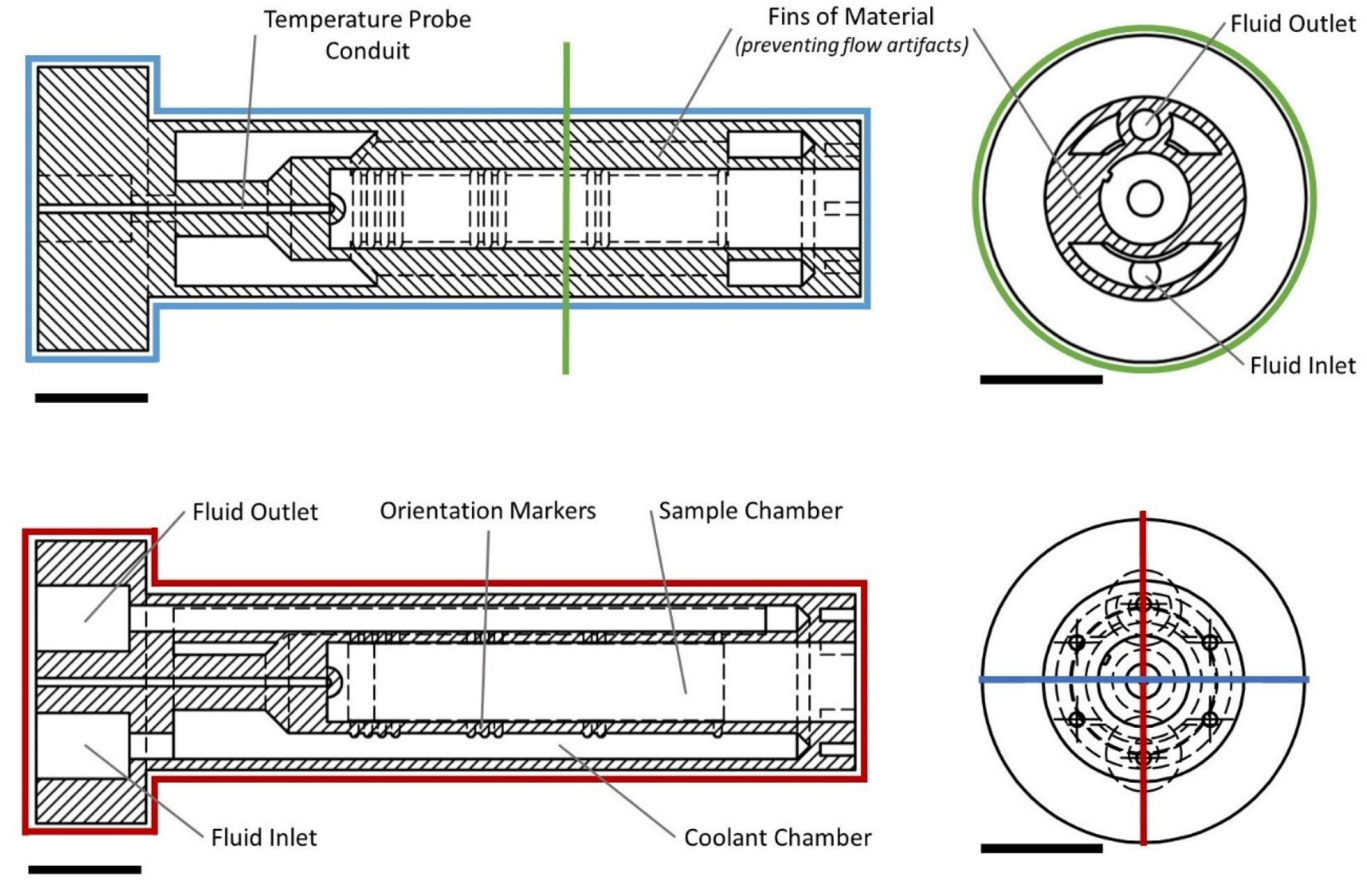
CAD drawings of cooling device for MRI imaging of frozen excised plant specimens. Longitudinal cutaway views at orthogonal planes; one view showing locations of material fins in the imaging plane (top image, blue line); and one view showing the inlet and outlet ports for the circulating coolant (bottom image, red line). Both longitudinal views show the temperature probe conduit for sample chamber temperature monitoring. Also shown is an axial cutaway view (green line). Orientation markers are indicated in each cutaway view. Bars equal 2 cm

The cooling device has several innovative design features that facilitated the necessary cooling of samples and the acquisition of quality images (Fig. 3). The resulting device had two chambers, an inner sample chamber surrounded by a coolant chamber through which chilled ethylene glycol flowed (Fig. 3). The coolant chamber was designed to envelop the sample chamber, facilitating cooling through convective heat loss from the samples as coolant flowed around them, as well as conductive heat loss through the barrier between the two chambers. Orientation markers were included around the sample chamber for longitudinal and axial sample positioning (Fig. 3, Bottom). The fluid inlet and outlet were tapped to accommodate commercial tubing connectors (Table 2).

**Fig. 4:**
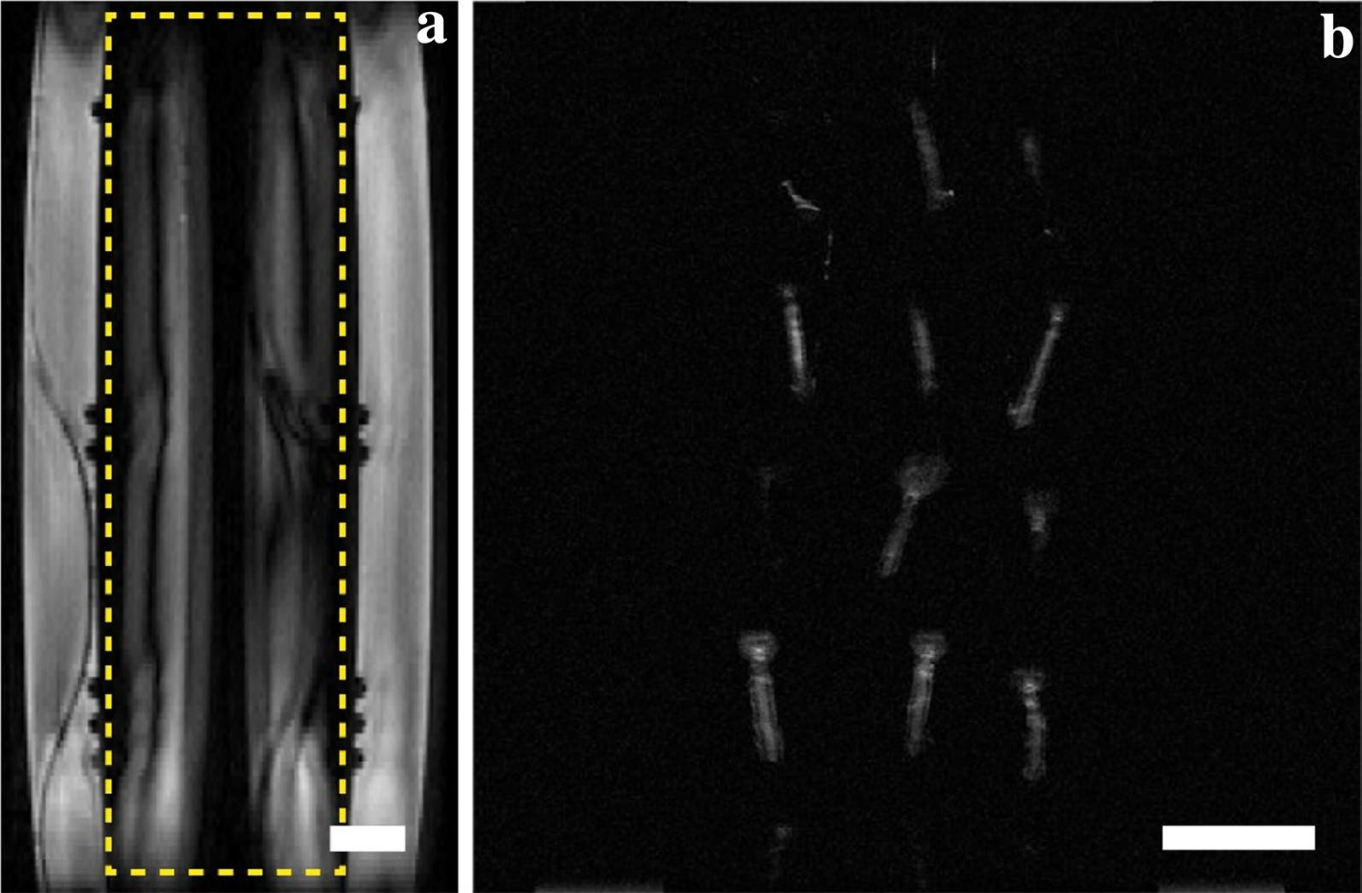
Imaging artifacts created by the flow of ethylene glycol in the coolant chamber. Imaging artifacts (within highlighted box) (**a**) were eliminated from the sample chamber view by modifying the device to include material fins to divert the coolant flow from the imaged volume along the longitudinal axis of the device. As a result, clear images of *V. macrocarpon* (**b**) bud samples were obtained. Scale bar equals 5 mm

Through several prototype iterations, we detected the need to include fins of material on either side of the sample chamber in the imaging plane (Fig. 3, Top) to remove imaging artifacts created by the flow of the circulating ethylene glycol, which obscured the view of the sample chamber (Fig. 4a). The inclusion of the fins directed the ethylene glycol outside of the imaging plane, allowing the clear imaging of the *V. macrocarpon* buds (Fig. 4b).

### Cooling calibration and frost control

A calibration test was performed to determine the variations in temperature between the set temperature on the circulating bath and the target temperature measured inside the device (Table 1). The calibration was necessary due to the lack of access to a MRI-compatible temperature probe able to monitor the freezing temperatures in real time. For the calibration, the experimental freezing rate of 3 °C/h was used, and once a test temperature was reached, it was held for 20 min to ensure equilibration. Once equilibrium was reached for a given test temperature, conditions in the bath and the device varied by less than 0.1 °C. As expected, temperatures in the cold bath were consistently lower than those measured inside the device, due to heat gain through the tubing that ran between the bath and the cooling device, despite two layers of insulation.

**Table 1:** Calibration of circulating coolant bath set temperatures to device experimental treatment temperatures

Cold bath set temperature (°C)	Corresponding device temperature (°C)
− 7.9	− 7.0 ± 0.1
− 16.0	− 14.0 ± 0.1
− 24.2	− 21.0 ± 0.1

Determination of correspondence of temperatures between the circulating coolant bath and the effective temperature experience at the device installed inside the MRI equipment. Temperature variations are attributed to coolant (ethylene glycol) heat gain during circulation through the tubing system

Frost control around the device was an important consideration during the experimental procedure, as excess moisture could damage the MRI system or the supporting electronics. This problem was compounded by the extended acquisition times (52 min) required for the MRI, as well as the slow freezing rates, which resulted in the system running for several hours at a time. The foam insulation covering the entire length of the tubing system limited frost buildup, as well as reduced heat transfer to the flowing coolant. A 3.5 mm thickness neoprene sleeve (Seattle Fabrics, United States) was also placed around the cooling device during scans to protect the coil and MRI components.

### *Vaccinium macrocarpon* bud freezing with our novel device

**Fig. 5:**
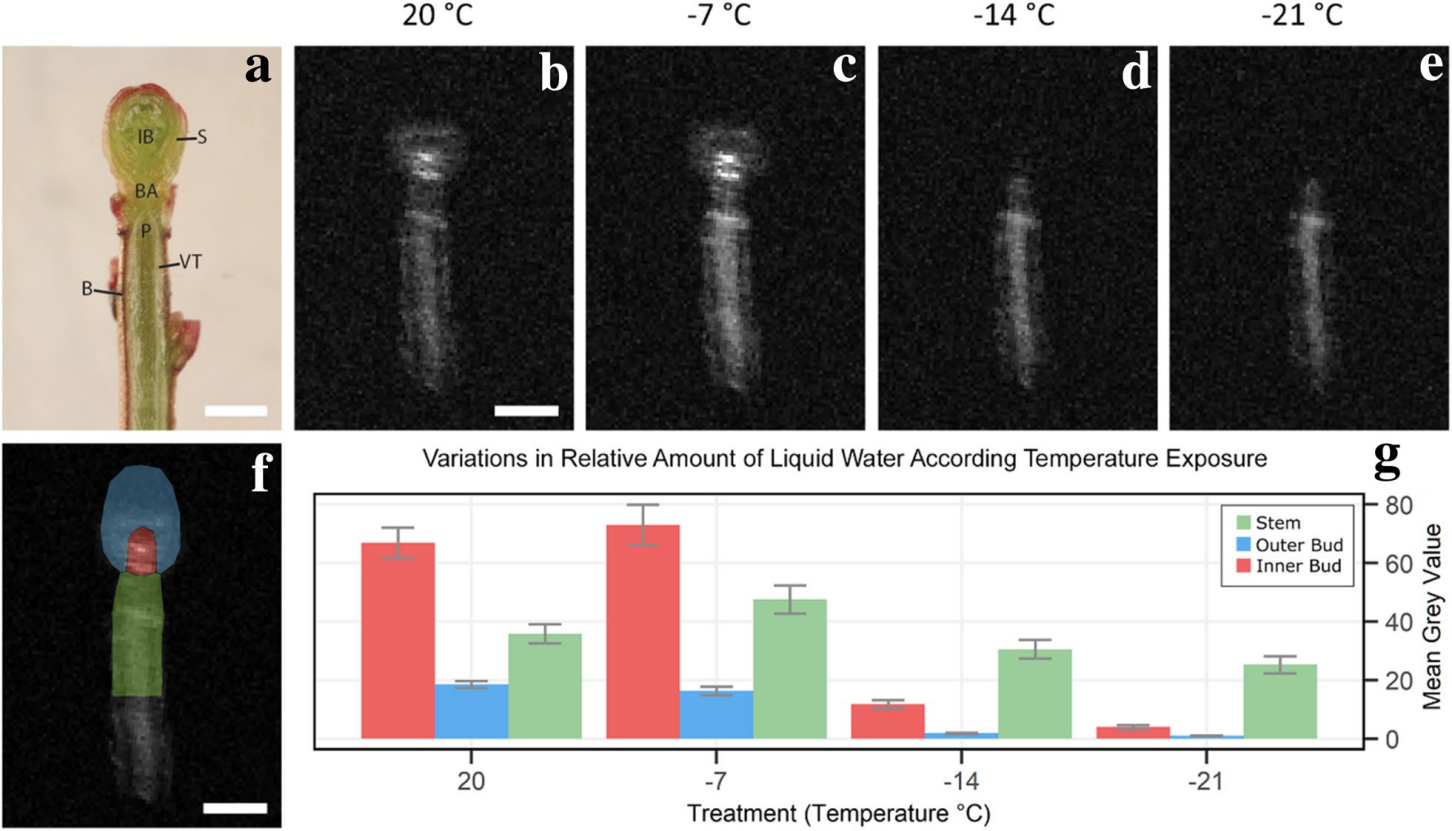
Example of a fresh *V. macrocarpon* bud in longitudinal section, obtained MRI images at evaluated temperatures, and plots of MGVs by defined ROIs. Fresh longitudinal section of *V. macrocarpon* bud indicating main anatomical structures (**a**) (B = bark; BA = bud axis; IB = inner bud (apical meristem and flower primordia); P = pith; S = bud scales; VT = vascular tissue). Longitudinal MR images obtained at 20, − 7, − 14, and − 21 °C (**b**–**e**, respectively) from specimens collected in November 2018 (n = 14). Images are close-ups of averages of 23 scans with a field of view of 30 × 30 mm with 512 × 512 pixels. Higher MGV (brighter) areas of images indicate unfrozen tissues. Evaluated ROIs of *V. macrocarpon* bud (**f**): blue = outer bud; red = inner bud; green = stem. Plot of changes in MGV for each ROI by temperature exposure (G), originally published in Villouta et al. [19]. Vertical error bars represent the standard error of the mean. Scale bar in A, B, and F equals 1 mm

Images of *V. macrocarpon* buds from the MRI scans did not have sufficient quality to identify individual tissues within the bud, as compared to a dissected sample (Fig. 5a to e), but this was expected due to the small bud size (1–2 mm diameter). However, the images had sufficient resolution to distinguish regions of interest (ROI) within the bud (i.e., outer bud, inner bud, and stem section) (Fig. 5f). Only buds yielding a complete longitudinal slice were selected (n = 14) for image analysis. Images were analyzed by determining the mean gray values (MGV) for the three ROIs: outer bud, inner bud, and stem (Fig. 5f) for each bud. MGV is a useful indicator of water and its physical state in these samples [9], where bright areas represent liquid water and dark areas represent frozen water. Variations in MGV in the ROI were observed over the range of experimental temperatures (Fig. 5g). The increase in average MGV as the samples cooled to − 7 °C is the result of an enhancement of signal detection as the sample and coil temperatures decrease [10] and is also an effect from a change in the Boltzmann distribution [16]. A rapid decrease in signal intensity from − 14 °C to − 21 °C is surmised to correspond with the temperature range over which liquid water in these regions of the bud tissue gradually freeze.

### Comparison to controlled freezing test (CFT) data

Near-simultaneously to the MRI acquisitions, samples were evaluated using a controlled freezing test (CFT), a conventional technique for cold hardiness assessments [17]. CFT involves placing the samples in a programmable freezer, where the air temperature decreases at a controlled rate. Following this, frozen samples are removed from the programmable freezer at pre-determined tested temperatures and placed on ice. This step allows samples to thaw at a slow rate for tissue recovery and damage expression to occur. Damage is scored through visual quantification of tissue browning and water-soaking appearance.

**Fig. 6:**
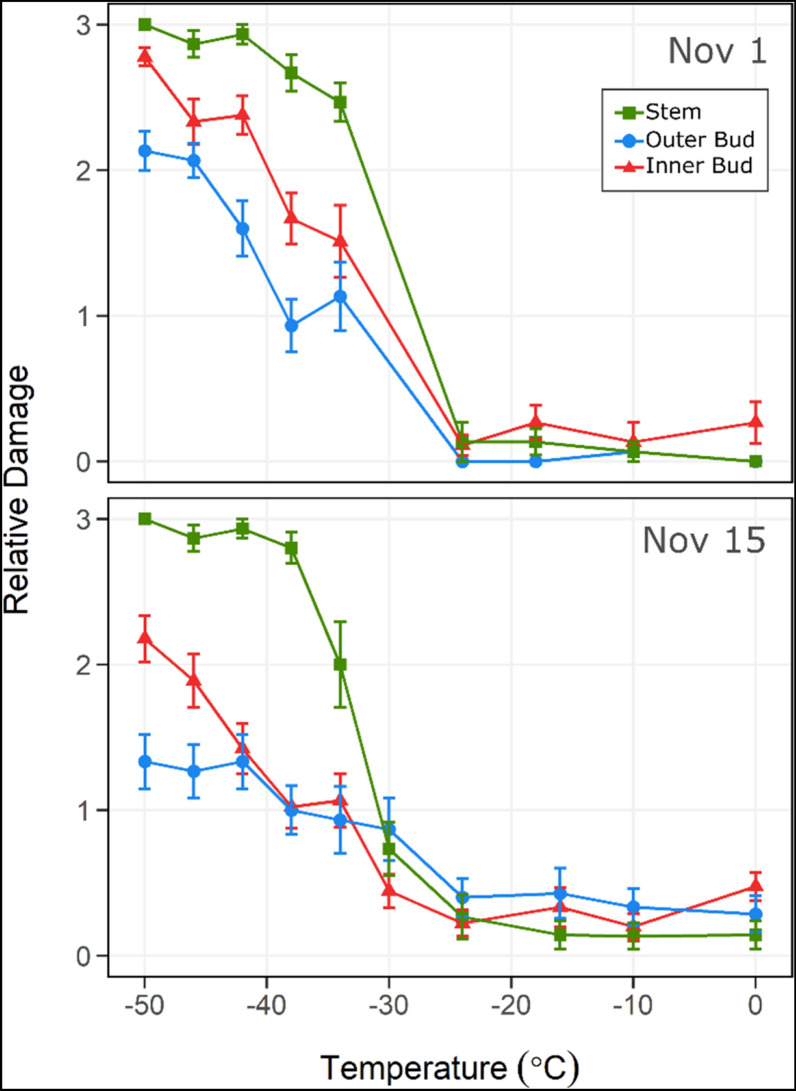
CFT-incurred damage in *V. macrocarpon* terminal buds. Plots of the mean relative damage to each of the three bud areas in terminal buds of *V. macrocarpon* by the degree of exposure to sequential temperatures (°C) in controlled freezing tests (CFTs). Buds were sampled November 1 and 15, 2018 from a commercial farm near Nekoosa, WI. Relative damage was scored using a discrete scale with four levels of severity, where 0 represents no damage and 3 complete damage (n = 15). Vertical bars represent the standard error of the mean

Freezing events detected from the MRI acquisitions were not detectable in the CFT observations as damaging. In the MRI, the outer bud MGV decreased to values near 0 at − 14 °C and − 21 °C (Fig. 5g), and this reduction in the relative content of liquid water was indicative of a freezing event [6]. However, in the CFT, the same ROI did not show damage (expressed as tissue browning) until temperatures reached below − 24 °C (Fig. 6). Similarly, a reduction in the inner bud MGV was observed at − 14 °C, with a larger decrease at − 21 °C (Fig. 5g). However, in the CFT, damage was noted only when temperatures were below − 24 °C (Fig. 6). Our results show that the use of the cooling device during MRI facilitates the detection of non-damaging freezing events in the inner structures of buds, which are not detected by the traditional CFT technique.

## Discussion

This device and accompanying system has the potential to reach temperatures lower than reported in this study. However, several factors must be considered in advance, such as the freezing capability of the coolant bath, the effectiveness of the tubing system insulation, and the viscosity of the coolant at freezing temperatures. Reaching lower temperatures could expand this device's use to the study of plant species with buds or other tissues with reported survival at temperatures lower than − 21 °C [18].

While this device represents an important development in plant imaging tools, there are several potential design and procedure improvements for future iterations. The incorporation of a MR-compatible temperature probe would permit real-time temperature measurements of the samples during image acquisition, rather than the reliance on the calibration data obtained pre-experiment with an incompatible temperature probe. Due to the toxicity of ethylene glycol, an alternative coolant, such as propylene glycol, could be considered to improve safety and ease of use. Since a key aspect of this device is the interchangeability of the three sample holders, additional holder designs could be made to expand the sizes of excised plant specimens tested. As well, improvements to the control of sample orientation within the tubes of the sample holder would increase the likelihood of capturing image slices along the longitudinal axes of the samples. Lastly, a custom plug to simultaneously cap all of the tubes of the sample holder would streamline preparation.

## Conclusion

This device, in conjunction with the circulating cooling system, allows researchers to successfully freeze samples in a stepwise way while performing MRI acquisitions. The low-cost of 3D printing for the main device components will lower the technical barrier to using MRI technology to visualize the freezing patterns of complex plant organs. Widespread use of this technique could further our knowledge of the mechanisms of freezing stress resistance in plants. All necessary design files for this device are made available as open-source at: https://morgridge.org/designs. It is the hope of the authors that other research groups will build this device and iterate upon it.

## Methods

### Experimental setup

This experimental setup is comprised of the cooling device and numerous pieces of supporting hardware (Fig. 2). Images of the fabricated device can be seen in Fig. 2a–c, including each component and two separate views of the assembled device. A coolant system pumps chilled coolant (ethylene glycol) from a circulating bath into the coolant chamber of the device surrounding the sample chamber, providing localized cooling. Clear high chemical resistance Tygon® R-3603 laboratory tubing (0.25 inch I.D., 0.378 inch O.D.) (Saint-Gobain, Malvern, PA) (Fig. 2d–e) is insulated and transports ethylene glycol (1:1 diluted) (Prestone, Lake Forest, IL) from a refrigerated circulating bath (Model AD07R-40, Polyscience, Niles, IL) via a Masterflex L/S Variable-Speed Console peristaltic pump (Cole-Palmer, Vernon Hills, IL) (Fig. 2f–i). Each line of the Tygon® tubing was wrapped with 1.5 mm thick single-side adhesive neoprene sheeting (The Felt Store, Ontario, Canada) and jointly covered by a 1.9 cm thick pre-slit polyethylene pipe insulation (Tundra, United States). A 3.5 mm thick neoprene (Seattle Fabrics, United States) sleeve was custom-made to jacket the device to inhibit frost formation and contribute insulation.

### Sample preparation

*Vaccinium macrocarpon* buds were collected from a commercial cranberry farm in Nekoosa (44° 16′46.9" N, 89° 55′00.4" W), WI, and sampled on November 7, 2018. Buds were collected and transported in sealed plastic bags on ice. Samples were processed within 24 h of field collection for MRI acquisition. *V. macrocarpon* uprights were sorted by terminal bud size, selecting for medium-size buds (1–2 mm diameter).

**Fig. 7:**
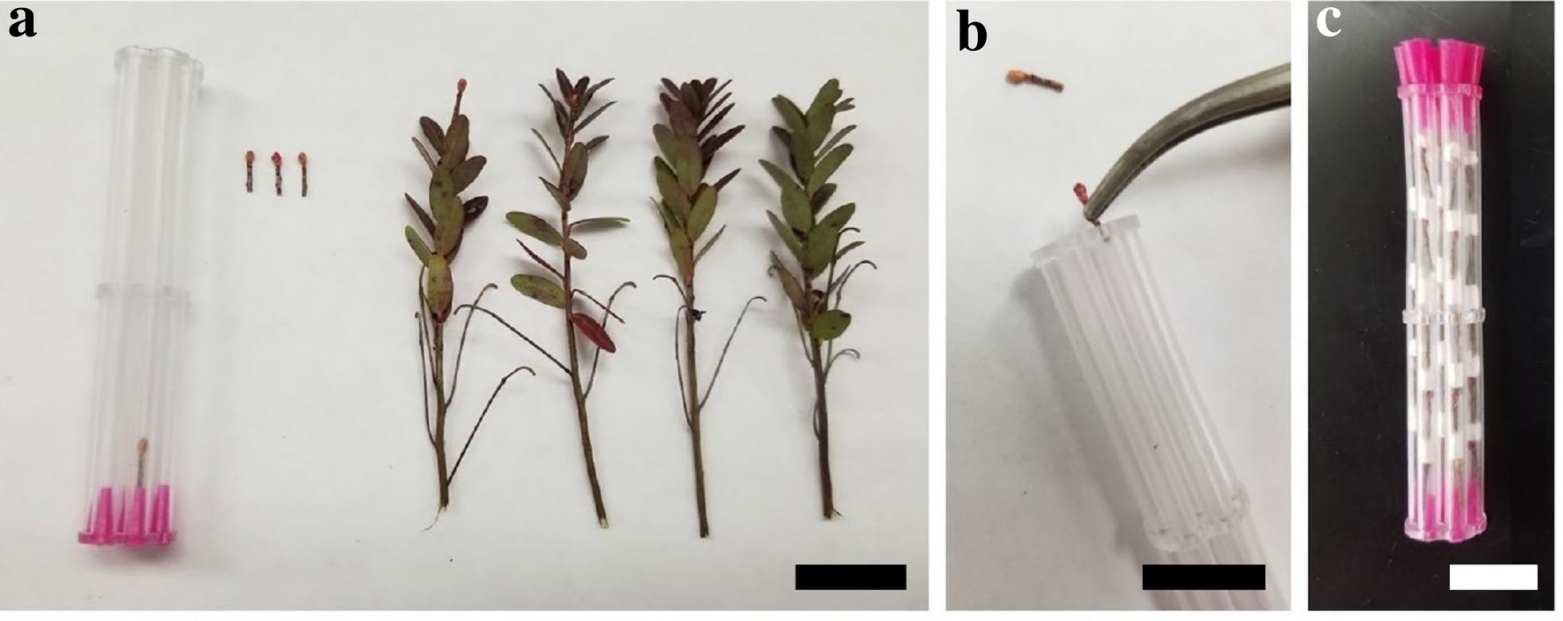
Loading of *V. macrocarpon* bud specimens into cooling device sample holders. *V. macrocarpon* samples prepared for controlled freezing MRI run, including untrimmed and trimmed buds (**a**), trimmed buds being loaded into tubes of the sample holder (**b**), and a loaded sample holder, with buds separated by paper stoppers (**c**). Bars equal 1 cm

*Vaccinium macrocarpon* terminal buds were excised leaving 5 mm of stem attached with no leaves (Fig. 7a). Cut ends of the stems were sealed with a layer of high vacuum grease (Dow Corning, Midland, MI) to avoid dehydration. Buds were lined up in the tubes of the smallest sample holder (Fig. 7b), separated by paper stoppers between buds (Fig. 7c). 28 buds were placed inside the sample holder. Once buds were positioned inside the sample holder, cylinder ends were capped with silicone plugs. Next, the sample holder was placed inside the sample chamber of the cooling device (Fig. 7c) and an inert perfluorocarbon (to enhance the MR image clarity) (Fluorinert, 3 M, St. Louis, MN) was poured into the interstitial space prior to sealing the device.

### Controlled freezing test

The CFT procedure was based on that of Villouta et al. [19], and was performed on two sampling dates, November 1 and 15, 2018. Both CFT tests were performed in a programmable freezer chamber (Tenney Model T2C, Thermal Product Solutions, New Columbia, PA, USA). Additional temperature monitoring was done with two copper-constantan (Type T) thermocouples (22 AWG) located inside the freezing chamber.

Uprights of at least 10 cm long and with a dormant tight bud were selected and rinsed in tap water. After trimming to 8 cm long, uprights were blotted dry with a paper towel. Uprights were grouped in bunches of five, and at their cut end, the set was wrapped with moist paper towel. Sets were placed in 50 ml plastic centrifuge tubes with plastic caps. Three replicate tubes were used at each test temperature. For the temperature lowering scheme, the first step was to reach − 1 °C and maintain it for one hour to allow thermal equilibrium. On following steps, decreasing rates were 1, 2, and 4 °C/h for the intervals − 1 to − 6 °C, − 6 to − 12 °C and − 12 °C to the minimum evaluated temperature, respectively. For ice nucleation induction, the racks containing the sample tubes were firmly shook during the temperature equilibration at − 1 °C.

Sets of three tubes were removed from the freezing chamber at eight test temperatures, ranging between 0 and − 50 °C. The removed tubes were placed in ice and darkness for 12 h followed by a period of 72 h at 4 °C in darkness to allow recovery of any potential tissue injury. Afterwards, tubes were held at room temperature for 24 h in low light conditions to allow damage symptom expression in bud structures.

### Bud damage evaluation

Bud damage evaluation was performed as described in Villouta et al. [19], using an Olympus SZX12 dissection microscope with a 1× objective (Olympus Optical Company, Tokyo, Japan) connected to a Canon EOS Rebel T6i digital camera (Canon U.S.A., Inc., Melville, NY, USA). For the evaluation of browning distribution and severity, buds were sectioned longitudinally with a razorblade and immediately assessed. The evaluated bud regions were determined based on the structures determined by Villouta et al. [19] where bud scales represent the outer bud, the shoot apical meristem (SAM), flower primordia, and bud axis comprised the inner bud, and the attached stem section represents the stem (Fig. 5f). A discrete scale comprising four levels of damage, ranging from 0 (no damage) to 3 (complete damage) in terms of the severity of oxidative browning and water-appearance, was used.

### Image acquisition

MRI was performed using a Varian 4.7-T small animal imaging system, and an Agilent/Varian ^1^H 200 MHz quadrature birdcage volume coil (Agilent Technologies, Palo Alto, CA, USA). The cooling device was inserted inside the coil for scanning. Images were acquired using a spin echo multi-slice (SEMS) pulse sequence. Acquisition parameters were set as follows: TR 306.4 ms, TE 21.5 ms, N 20, 512 × 512 matrix. Field of view was 30 × 30 mm with 10, 1-mm thick slices scanned 23 times. The MRI run consisted of equilibration of the sample chamber for 20 min at each treatment temperature and a hold for the duration of each image acquisition. Total acquisition time per test temperature was 52 min. After the 20 °C image acquisition, the chamber temperature was ramped to − 1 °C at a rate of 10 °C/h and held for 1 h. Subsequently the rate of cooling between each treatment temperature was 3 °C/h. Evaluated temperatures were 20, − 7, − 14 and − 21 °C and were achieved by manually adjusting the circulating bath temperature set point per the earlier performed calibration (Table 2).

**Table 2:** Parts list for cooling device and supporting equipment

Description	Manufacturer/supplier	Part #
Tubing	Fisher Scientific Saint-Gobain	14-169-1J
Inner insulation	Amazon (The Felt Store)	F-INVNEOADH-1
Outer insulation	Tundra	6XP068038
Neoprene sleeve	Seattle Fabrics	N3-2
Quick Connectors	McMaster-Carr	9198T16, 9198T18
Pump	Cole-Palmer Instruments Co	EW-77910-40
Circulating bath	Polyscience	AD07R-40
Coolant	Prestone	AF2000
Sample plugs	McMaster-Carr	9277K36 (e.g.)
Fluorinert (for sample chamber)	3 M	FC-3283
Vacuum grease	Variety Available	N/A
10–32 Nylon Bolt	McMaster-Carr	94320A430
Bolts 2	McMaster-Carr	95868A258
Small O-Ring	McMaster-Carr	9452K17
Large O-Ring	McMaster-Carr	1288N116
Device main body	Custom	N/A
Device cap	Custom	N/A
Sample holders	Custom	N/A

Cooling device parts and accessories, including manufacturer/supplier and part number. Custom pieces were designed and 3D printed; design files are available for download (in STL format) at https://morgridge.org/designs

### Temperature calibration

Since we lacked access to a MRI-compatible temperature probe able to measure freezing temperatures, the device sample holder temperature could not be actively monitored during the imaging runs. A calibration test was required to determine the sample temperature inside the cooling device relative to that reported by the circulating bath before imaging acquisition could occur. For this purpose, a central isolated conduit was included in the design of the device (Fig. 3) for the insertion of a non-MRI compatible copper-constantan (Type T) micro-thermocouple (36 AWG). This conduit allowed the junction of the thermocouple to reach the base of the sample chamber. The device was placed in the coil and bore of the MRI system to approximate the environment during data collection. Appropriate set points on the circulating bath were determined to reach the desired experimental temperatures.

### Data analysis

Image files were viewed with Multi FDF Opener, a plugin of ImageJ, an open-source image analysis software package (NIH) [20]. Images were normalized to the same window and level for comparing contrast between the different proton signal intensities for each of the evaluated temperatures. Image processing was performed using ImageJ. ROI were drawn on normalized images of individual buds based on features of *V. macrocarpon* anatomy. MGV were evaluated within the ROI and compared across all temperature treatments (Fig. 5g). The intensity of pixels decreased with freezing of the liquid water inside the bud tissues.

## Data Availability

The designs files for the device presented here are available from the Morgridge Institute for Research repository at: https://morgridge.org/designs.

## Abbreviations

MRI: Magnetic resonance imaging
MGV: Mean gray values
ROI: Region of interest
CFT: Controlled freezing test
